# Evodiamine, a Novel NOTCH3 Methylation Stimulator, Significantly Suppresses Lung Carcinogenesis *in Vitro* and *in Vivo*

**DOI:** 10.3389/fphar.2018.00434

**Published:** 2018-05-01

**Authors:** Tao Su, Xia Yang, Jian-Hua Deng, Qiu-Ju Huang, Su-Chao Huang, Yan-Min Zhang, Hong-Ming Zheng, Ying Wang, Lin-Lin Lu, Zhong-Qiu Liu

**Affiliations:** International Institute for Translational Chinese Medicine, Guangzhou University of Chinese Medicine, Guangzhou, China

**Keywords:** evodiamine, lung cancer, Notch3, NOTCH3 methylation, DNA methyltransferases

## Abstract

Lung cancer is a leading cause of cancer-related deaths worldwide. NOTCH3 signaling is mainly expressed in non-small cell lung carcinoma (NSCLC), and has been proposed as a therapeutic target of NSCLC. While, few agents for preventing or treating NSCLC via targeting NOTCH3 signaling are used in modern clinical practice. Evodiamine (EVO), an alkaloid derived from Euodiae Fructus, possesses low toxicity and has long been shown to exert anti-lung cancer activity. However, the underlying anti-lung cancer mechanisms of EVO are not yet fully understood. In this study, we explored the involvement of NOTCH3 signaling in the anti-lung cancer effects of EVO. Urethane-induced lung cancer mouse model and two NSCLC cell models, A549 and H1299, were used to evaluate the *in vivo* and *in vitro* anti-lung cancer action of EVO. A DNA methyltransferase inhibitor was employed to investigate the role of NOTCH3 signaling in the anti-lung cancer effects of EVO. Results showed that EVO potently reduced tumor size and tumor numbers in mice, and inhibited NOTCH3 in the tumors. EVO also dramatically reduced cell viability, induced G2/M cell cycle arrest, inhibited cell migration and reduced stemness in cultured NSCLC cells. Mechanistic studies showed that EVO potently inhibited NOTCH3 signaling by activation of DNMTs-induced NOTCH3 methylation. Importantly, inhibition of NOTCH3 methylation in NSCLC cells diminished EVO’s anti-NSCLC effects. Collectively, EVO, a novel NOTCH3 methylation stimulator, exerted potent anti-lung cancer effects partially by inhibiting NOTCH3 signaling. These findings provide new insight into the EVO’s anti-NSCLC action, and suggest a potential role of EVO in lung cancer prevention and treatment.

## Introduction

Lung cancer is one of the most prevalent cancer types worldwide, with approximately 1.8 million newly diagnosed cases every year (Torre et al., 2015). It is generally consisting of two subtypes: non-small cell lung cancer (NSCLC) and small cell lung cancer (SCLC) (Zou et al., 2015). NSCLC is a major form of lung cancer, which accounts for 80–85% of the total cases (Tong et al., 2016). Most NSCLC patients are diagnosed at an advanced stage, only 10–20% of the patients could be diagnosed at early stage. Although there are a variety of available therapies for NSCLC treatment, the prognosis for NSCLC is still very poor. The 5-year survival rate in advanced NSCLC is no more than 20% (Xia et al., 2017). Most of the chemotherapeutics have failed in NSCLC treatment due to their limitations, e.g., drug resistance, high toxicities, and so on (Alberg et al., 2013; Tong et al., 2016). Targeted therapy of lung cancer is the most developed field of cancer treatment. At present, molecule targeted therapy has achieved great progress in NSCLC because of the better understanding of the mechanisms of tumorigenesis and signal transduction pathway as well as the metabolic process in lung cancer. For example, inhibitors of epidermal growth factor receptor (EGFR) and anaplastic lymphoma kinase (ALK) have been successfully utilized in the treatment of NSCLC. But unfortunately, almost all of the patients would face drug resistance ultimately, therefore, finding new therapeutic targets become the research focus.

Many signaling pathways including Notch signaling are involved in lung cancer development and progression. Notch receptors (Notch 1–4) are transmembrane proteins which are activated after binding with ligands (Jagged and Delta-like families). Upon binding, Notch receptor undergoes a serious of proteolytic cleavages, leading to the release of Notch intracellular domain (NICD), that translocates to the nucleus and regulates the downstream targets expression, e.g., c-Myc, Hey-1, and Hes1 (Zou et al., 2018). Constitutive active NOTCH3 is frequently detected in NSCLC, and is considered as a pathogenic biomarker and a therapy target for NSCLC (Li et al., 2011). Inhibition of NOTCH3 using some small molecular inhibitors can restrain tumor growth and induce cell apoptosis (Konishi et al., 2007, 2010). Thus, NOTCH3 is an important therapeutic target in lung cancer. Moreover, chemotherapy targeting NOTCH3 should be able to assault lung cancer on multiple fronts. Abnormal DNA methylation is a common phenomenon in malignant tumors. The hallmark of cancer epigenetics is aberrant DNA methylation, which consisting of regional hypermethylation of tumor suppressor genes and hypomethylation of oncogenic genes by DNA methyltransferases (DNMTs) (Rajendran et al., 2011). Detection of the level of DNA methylation (e.g., NOTCH3 methylation) is expected to be an important approach for early diagnosis of lung cancer (Zhang et al., 2017).

Evodiamine (EVO) is one of the major bioactive constituents that isolated from the herb *Euodiae Fructus* (Chinese name, *Wu-Zhu-Yu*). It has multiple biological functions including anti-inflammatory (Fan et al., 2017), anti-obesity (Tan and Zhang, 2016), neuroprotective (Huang et al., 2014) and anti-cancer activities (Zhao et al., 2015; Zhong et al., 2015). It shows cytotoxic effects on several types of cancer cells, e.g., breast cancer cells (Du et al., 2013), gastric cancer cells (Shen et al., 2015), bladder cancer cells (Zhang et al., 2014), and lung cancer cells (Lin et al., 2016). In lung cancer cells, EVO has been found to be able to inhibit cell proliferation (Lin et al., 2016), induce cell apoptosis (Mohan et al., 2016), suppress cell migration and invasion (Ogasawara and Suzuki, 2004), and induce reactive oxygen species (ROS) production (Yang et al., 2008). However, up to now, the anti-lung cancer mechanisms of action of EVO are poorly understood. Here, we evaluated the *in vivo* and *in vitro* anti-lung cancer effects of EVO, and examined whether NOTCH3 signaling is involved in the EVO’s anti-cancer action. Two human NSCLC cell lines with constitutively active NOTCH3, A549 and H1299 (Hassan et al., 2016), and the urethane-induced lung cancer mice model were employed in this study.

## Materials and Methods

### Chemicals and Reagents

Evodiamine (purity > 98%) was purchased from Dalian Meilun Biotech Co., Ltd. (Dalian, China). 3-(4,5-dimethylthiazol-2-yl)-2,5-diphenyltetrazolium bromide (MTT), 5-aza-2′-deoxycytidine (5-aza) and trichostatin (TSA) were purchased from Sigma-Aldrich (St. Louis, MO, United States). Propidium iodide (PI) staining, CD44 and CD133 were purchased from Biosciences (BD Biosciences, NJ, United States). β-actin, E-cadherin, N-cadherin and Vimentin primary antibodies were purchased from Santa Cruz Biotechnology (Santa Cruz, CA, United States). cdc2, cyclinB1, DNMT1, DNMT3A, DNMT3B, Histone H3 primary antibodies and HRP-conjugated secondary antibody were obtained from Cell Signaling Technology Inc. (Beverly, MA, United States). Notch3 primary antibody was purchased from Abcam (Cambridge, United Kingdom).

### Animals and Treatments

Female FVB mice (20–22 g) were purchased from Laboratory Animal Center of Sun Yat-sen University [License number: SCXK (Guangdong) 2016-0029; Guangzhou, China], and kept in the animal facility in the SPF animal laboratory [License number: SYXK (GZ) 2014-0144] at International Institute for Translational Chinese Medicine, Guangzhou University of Chinese Medicine (Guangzhou, China). Animal experiments were approved by the Guangzhou University of Chinese Medicine Animal Care and Use Committee (Guangzhou, China), and conducted according to the ethical standards and national guidelines. Mice were injected intraperitoneally with urethane (600 mg/kg) weekly for 15 weeks (Ma et al., 2016), then they were randomly divided into three groups of 10 each. Mice were then intragastrically (i.g.) administered with 0.5% CMC-Na solution (vehicle), 5 and 10 mg/kg of EVO for 22 weeks (5 times per week), respectively. To monitor the toxicity of EVO, general clinical observations (e.g., changes in eyes, fur, skin, excretions and autonomic activity) were made once a day. Changes in gait, posture or bizarre behavior were also recorded. Body weight of each mouse was measured once a week. At the end of the experimental period, mice in each group were euthanized by CO_2_ asphyxiation. Organs (including liver, kidney, heart, spleen, and thymus) of each mouse were dissected and weighed. The tumor number and tumor volume (TV) of lung tissues were measured. TV, defined based on dimension (D), was calculated as formula: TV (mm^3^) = D^3^/2.

### Hematoxylin and Eosin (H&E) Staining

Lung tissues were dissected. Half of the tissues were fixed in 4% paraformaldehyde, embedded in paraffin, and then sliced up (4 μm). Following, the slices were stained with hematoxylin and eosin, and examined by light microscopy (Wu et al., 2017).

### Immunohistochemistry

Slices (4 μm) of lung tissue were prepared as described in Section “H&E Staining,” and then all slices were dewaxed, hydrated and incubated with sodium citrate for antigen retrieval. After that, they were rinsed with phosphate buffered saline (PBS), and incubated with diluted anti-Notch3 antibody overnight at 4°C. Slices were then stained using a immunostaining kit (BOSTER Biological Technology) followed by the manufacturer’s instructions.

### Analysis of NOTCH3 Alterations in Lung Cancer Patients

The expression and methylation of NOTCH3 in human normal lung tissues and human lung tumor tissues were checked and analyzed in TCGA from MethHC database^1^. The survival analysis was conducted in 3,021 lung cancer patients using Kaplan–Meier plotter^2^. NOTCH3 (203238_s_at) was entered as an input.

### Cell Culture

Two human NSCLC A549 and H1299 cell lines, human embryonic fibroblast MRC-5 cell line and the human bronchial epithelial BEAS.2B cell line were purchased from the ATCC (Manassas, VA, United States). Cells were cultured in RPMI-1640 (Gibco, Grand Island, NY, United States) supplemented with 10% FBS (GIBCO, United States) and 1% penicillin/streptomycin in a humidified 5% CO_2_ atmosphere at 37°C.

### MTT Assay

The cytotoxic effects of EVO on NSCLC (A549 and H1299) cells and normal embryonic fibroblast (MRC-5) cells were determined by the MTT assay (Liu et al., 2016). Cells were treated with EVO (0–40 μM) or vehicle for 48 or 72 h. The IC_50_ value of EVO was calculated using the GraphPad Prism software (version 5.0, CA, United States) for each cell line.

### EdU Assay

H1299 cells were seeded in 96-well plates (2 × 10^3^ cells/well). After 48-h treatment with EVO (1, 2, and 4 μM) or vehicle, cells were incubated with EdU labeling medium (50 μM) at 37°C for 2 h, fixed with 4% paraformaldehyde (pH 7.4) for 30 min, and then incubated with glycine for another 5 min. Then cells were incubated with anti-EdU working solution for 30 min and then stained with DAPI (1×) for another 30 min at room temperature. The images were captured using Leica DMI3000B (Leica, GER).

### Western Blotting

A549 and H1299 cells were seeded in 6-well plates (2.5 × 10^5^ cells/well) and treated with EVO (4, 8, and 16 μM for A549 cells and 1, 2, and 4 μM for H1299 cells, respectively) for 48 h. Animal tissues (normal and tumor) were rinsed with PBS for three times. Cell and tissue extracts were prepared using RIPA lysis buffer and phenylmethanesulfonyl fluoride (PMSF), and quantified using Coomassie Brilliant Blue Kit (Bio-Rad, Hercules, CA, United States). Then, protein samples were separated by 8, 10, and 12% SDS–PAGE, transferred to PVDF membrane, and blocked with 5% skimmed milk, and then incubated with the primary and secondary antibodies. ECL chemiluminescence reagent was applied to detect for fluorescent signals using FluorChem E (Santa Clara, CA, United States) (Wang et al., 2018).

### Real-Time Quantitative Polymerase Chain Reaction (PCR) Analysis

A549 and H1299 cells were seeded as described in Section “Western Blotting.” After 24-h treatment, total mRNA was isolated using TRIzol reagent (Invitrogen, United States) and reverse-transcripted into cDNA following the PrimeScript^TM^ RT reagent Kit (TaKaRa, Shiga, Japan). SYBR Green real-time PCR amplification and detection were then performed using an ABI 7500 system (Applied Biosystems, Foster City, CA, United States). Relative gene expression was normalized to GAPDH (Su et al., 2017). Sequences of PCR primers were shown in **Supplementary Table 1**.

### Immunofluorescence

A549 and H1299 cells were seeded in 15 mm confocal dish (3 × 10^4^ cells/well) and allowed to grow overnight, and then cells were treated with vehicle or EVO (4, 8, and 16 μM for A549 cells and 1, 2, and 4 μM for H1299 cells, respectively) for 48 h. After harvested, cells were fixed in 4% cold paraformaldehyde, and then incubated with Notch3 primary antibody (1:1000, v/v) at 4°C overnight. After washing with PBS, cells were incubated with Alexa Fluor^®^ conjugated secondary antibodies (1:500, v/v) at room temperature for 1 h (Lv et al., 2017). Images were captured using Leica TCS SP8 confocal microscope (Leica, GER).

### Methylation-Specific PCR (MSP)

A549 and H1299 cells were treated as previously described in Section “Western Blotting.” DNA was extracted using a PureLink^®^ Genomic DNA Mini Kit (Invitrogen, United States). Then, DNA was conducted CT conversion quantified using the MethylCode Bisulfite Conversion Kit (Invitrogen, United States) following the manufacturer’s instructions. agarose electrophoresis was used for separating the PCR products, and Quantity One software (Bio-Rad, CA, United States) was used for analyzing the bands (Lu et al., 2018). Sequences of PCR primers were shown in **Supplementary Table 1**.

### DNA Extraction and Preparation for MeDIP-Seq

A549 and H1299 cells were seeded in 25ml-flask at 1 × 10^6^ cells/flask, and treated with EVO (4, 8, and 16 μM for A549 cells and 1, 2, and 4 μM for H1299 cells, respectively) for 48 h. The DNA of cells were isolated using PureLink^®^ Genomic DNA Mini Kit (Invitrogen, United States) followed by the manufacturer’s protocols. Then, DNA was conducted CT conversion quantified according to MethylCode Bisulfite Conversion Kit (Invitrogen, United States). DNA libraries were sequenced using the Illumina Hiseq 2000 by Beijing Genomics Institute (Shenzhen, China). Briefly, after RNA transcription and uracil-specific cleavage by RNase A, MALDI-TOF MS was performed to analyze the cleavage products (methylated and non-methylated template DNA) (Sant et al., 2012; Lu et al., 2018).

### Cell Cycle Analysis

H1299 cells were treated with EVO (1, 2, and 4 μM) for 48 h, and then all cells were collected, fixed with ice-cold 70% ethanol at 4°C overnight, and stained with PI (BD Biosciences, San Diego, CA, United States) for 15 min. Stained cells were analyzed using a FACS AriaIII (BD Biosciences), and data were analyzed by FlowJo 7.6 software.

### Wound Healing Assay

H1299 cells were seeded into 6-well plates at 1 × 10^6^ cells/well and cultured overnight. Wounds were created using a plastic pipette tip. After washing with PBS, cells were treated with vehicle or EVO (1, 2, and 4 μM) in serum-free RPMI-1640 medium. Images of wounds were photographed at 0, 12, and 24 h using the Leica DMI3000B at 10 × magnification, and wound sizes at different time points were measured using the Image ProPlus 6.0 software.

### Soft Agar Colony Formation Assay

H1299 cells were suspended in medium containing 0.4% agar, and then added into 6-well plates filled with 1% agar (1000 cells/well). After treated with EVO (1, 2, and 4 μM) or vehicle for 2 weeks, colonies were counted and photographed.

### CD44 and CD133 Detection

The expression levels of cancer stem cell markers CD44 and CD133 were detected using FACS AriaII followed by the manufacturer’s instructions. Cells were seeded in 6-well plates (2.5 × 10^5^ cells/well) and treated with EVO (1, 2, and 4 μM) for 48 h. Then cells were collected, rinsed, and suspended in 500 μL PBS with 20 μL CD44 or CD133 antibody for 30 min. After that, the fluorescence was monitored by flow cytometry.

### Statistical Analysis

Data were presented as mean ± SD from three independent experiments. Statistical analysis was performed by one-way analysis of variance (ANOVA) followed by the LSD *post hoc* test using SPSS 19.0 software. Statistical analyses were carried out using GraphPad Prism (version 5.0, CA, United States). *p* < 0.05 was considered statistically significant.

## Results

### EVO Restrained Tumor Growth in Urethane-Induced Lung Cancer Mouse Model

**FIGURE 1:**
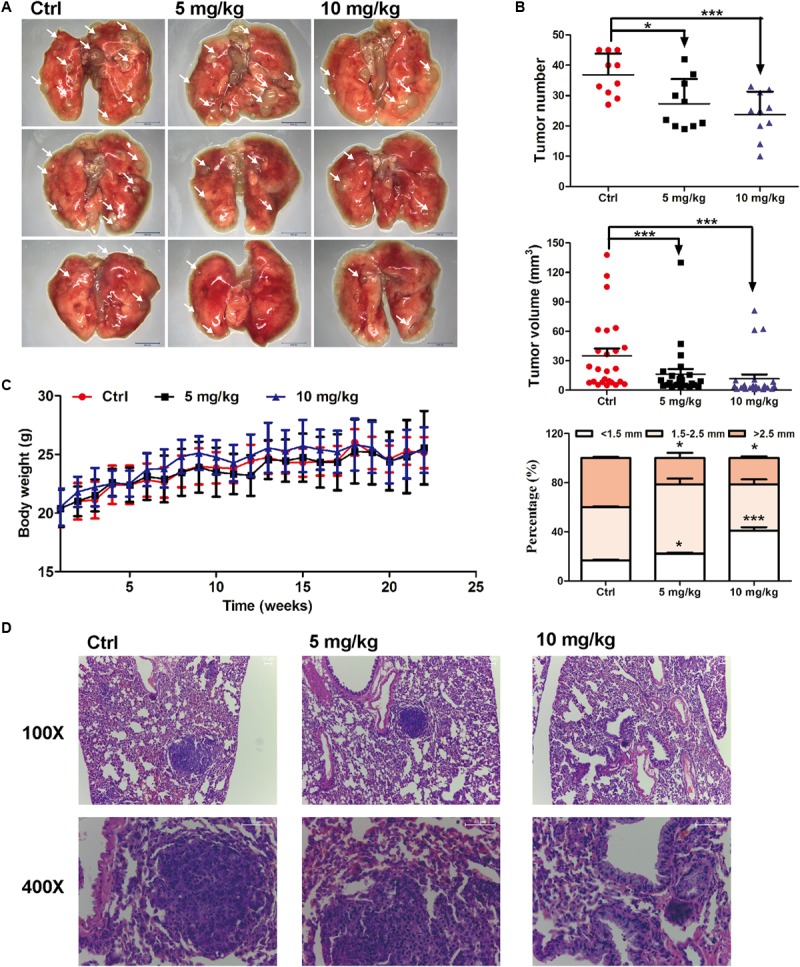
Anti-lung cancer effects of EVO in mice. **(A)** The photo of pulmonary nodules dissected from the FVB mice (scale bar: 10 mm). All photos were photographed using a stereoscopic microscope. **(B)** The tumor number, tumor volume and the percentage of different tumor size were shown (*n* = 10). **(C)** Body weights at different time points. In **(B)** and **(C)**, data were presented as mean ± SD of ten mice. **(D)** Representative H&E staining images of lung tissues. Tumors were photographed under a fluorescence inversion microscope (scale bar: 50 μm). ^∗^*p* < 0.05, ^∗∗∗^*p* < 0.001, *vs.* control group.

The *in vivo* anti-lung cancer effect of EVO was evaluated using a urethane-induced lung cancer mouse model (Ma et al., 2016). As shown in **Figures 1A,B**, i.g. 5 or 10 mg/kg of EVO for 22 weeks significantly inhibited tumor growth in mice, especially decreased the number of large tumor nodules (>2.5 mm). Compared with control group, the average tumor number in 5 mg/kg and 10 mg/kg of EVO-treated groups were remarkably reduced by 28.57% and 34.50%, respectively (**Figure 1B**). H&E staining results showed that well-defined focal lesions with marked cellular atypia and pleomorphisms were observed in control group, while, the focal lesions in EVO-treated groups were smaller compared with that in control group (**Figure 1D**). No changes of the organ (heart, liver, spleen, lung, kidney, and thymus) indexes were found in all mice (**Supplementary Figure 1**). The body weight of mice did not have significant difference among the three groups (**Figure 1C**).

### EVO Exhibited Higher Cytotoxicity in NSCLC Cells Than in Normal Embryonic Fibroblast Cells

**FIGURE 2:**
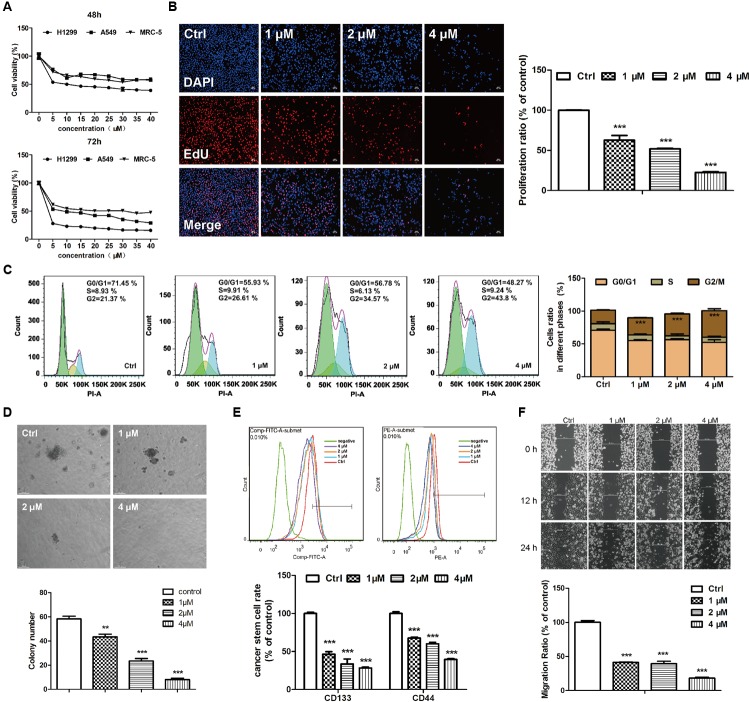
EVO exerted anti-lung cancer effects *in vitro*. **(A)** Cell viability was determined by the MTT assay. A549, H1299 or MRC-5 cells were treated with various concentrations of EVO (0–40 μM) or vehicle for 48 and 72 h, respectively. **(B)** H1299 cells were treated with vehicle or EVO (1, 2, and 4 μM) for 48 h. Cells were fixed, incubated with anti-EdU working solution, and then stained with DAPI. The images were captured using Leica3000B (Leica, GER). Representative images (left) and relative fluorescent levels (right) were shown. Fluorescent level of the vehicle-treated cells was regarded as 1. **(C)** H1299 cells were treated as previously described in **(B)**. After fixation, cells were stained with PI and then analyzed using a flow cytometry. Representative images (left) and the statistical analysis of cell cycle data in three independent experiments (right) were shown. **(D)** Representative images of colony formation were captured after treatment with EVO (1, 2, and 4 μM) for 2 weeks (scale bar: 100 μm), and the quantification of colony numbers were shown. **(E)** Cells were treated with vehicle or EVO (1, 2, and 4 μM) for 48 h, then the cells were collected and detected by flow cytometry. Representative images and relative expression levels were shown. Expression level of the vehicle-treated cells was regarded as 100%. **(F)** H1299 cells were plated in 6-well plates, and 1 day later, wounds were created in the confluent monolayer followed by vehicle or EVO (1, 2, and 4 μM) treatment, each scratch was photographed after 12- and 24-h treatments. Data were presented as mean ± SD from three independent experiments,^∗∗^*p* < 0.01, ^∗∗∗^*p* < 0.001, *vs.* vehicle.

The cytotoxicities of EVO were examined in two NSCLC (A549 and H1299) cell lines and a normal human embryonic fibroblast (MRC-5) cell line by using the MTT assay. Results showed that EVO reduced A549 and H1299 cell viabilities in both time- and dose-dependent manners (**Figure 2A**), with IC_50_ values of 41.13 μM and 12.43 μM after 48-h treatment, respectively. Furthermore, the cytotoxicity of EVO in MRC-5 cells was less potent than that in A549 and H1299 cells. In addition, in contrast to the abundant EdU-positive cells observed in control, EVO treatments significantly suppressed H1299 cells proliferation (**Figure 2B**).

### EVO Induced G2/M Cell Cycle Arrest in H1299 Cells

**Figure 2C** showed the quantified cell distributions in different phases. EVO treatments dose-dependently increased the percentage of H1299 cells in the G2/M phase from 21.37% ± 0.69% in control to 26.61% ± 0.57%, 34.57% ± 1.20% and 43.8% ± 2.90% in three concentrations of EVO treatments, respectively. The cyclin B-cdc2 complex has a critical role in the G2/M phase transition (Zhang et al., 2011). As demonstrated by Western blot analysis (**Supplementary Figure 2A**), EVO treatment dose-dependently decreased the protein levels of cyclin B1 and cdc2. These results suggested that EVO induced G2/M phase arrest in NSCLC cells.

### EVO Reduced Stemness of H1299 Cells

Soft agar colony assays were employed to explore EVO’s effects on cancer cell stemness. As shown in **Figure 2D**, EVO significantly lowered the colony number and reduced the colony size in H1299 cells. Next, we determined the expressions of CD44 and CD133 (two potential markers for identifying lung cancer stem cells) (Wang et al., 2012) in H1299 cells by flow cytometry. After 1, 2, and 4 μM of EVO treatments, CD44 positive cells were decreased from 45.47% ± 1.01% to 30.80% ± 0.57%, 27.23% ± 0.92% and 18.03 ± 0.47%, respectively; CD133 positive cells were decreased from 51.30% ± 0.85% to 23.87% ± 1.64%, 17.27% ± 3.35% and 14.53 ± 0.65%, respectively (**Figure 2E**).

### EVO Inhibited NSCLC Cell Migration

Wound healing assay was used for determining the effects of EVO on H1299 cells migration. After 24-h treatment, EVO dose-dependently inhibited H1299 cell migration (**Figure 2F**) (under all the conditions, EVO did not affect cell viability). Epithelial to mesenchymal transition (EMT) is the first step in the cascade of steps involved in tumor invasion and metastasis, which is associated with cancer metastasis and progression (Yeung and Yang, 2017). Consistently, the protein levels of EMT-related markers N-cadherin and vimentin were significantly reduced by EVO, whereas, the levels of E-cadherin were increased after EVO treatment in H1299 cells (**Supplementary Figure 2B**).

### High Expression of NOTCH3 Caused Poor Lung Cancer Prognosis

**FIGURE 3:**
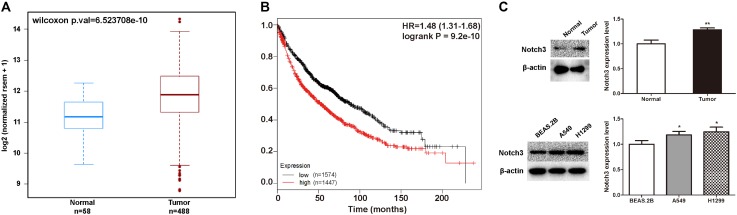
The expression of NOTCH3 in human lung tumors and cultured cells. **(A)** The expression of NOTCH3 in human normal lung tissues and lung tumor tissues were checked and analyzed in TCGA from MethHC database (http: //maplab.imppc.org/wanderer/). The dataset includes 488 cases of lung cancer patients and 58 cases of normal persons. **(B)** Kaplan–Meier analysis of the overall survival time of lung adenocarcinoma patients with high and low expressions of NOTCH3. **(C)** Normal lung tissues, pulmonary nodules, normal human bronchial epithelial cells (BEAS.2B) and NSCLC cells (A549 and H1299) were extracted for Western blot analysis by using antibody specific to Notch3, respectively. Data were presented as mean ± SD from three independent experiments, ^∗^*p* < 0.05, ^∗∗^*p* < 0.01, *vs.* normal lung tissues or normal bronchial epithelial cells.

Studies suggest that NOTCH3 is mainly expressed in NSCLC, and has been proposed as a therapeutic target of lung cancer. Here, we analyzed the relationship between NOTCH3 and lung cancer using TCGA, Kaplan–Meier and MethHC databases. TCGA analysis showed that NOTCH3 was highly expressed in lung adenocarcinoma patients (**Figure 3A**), which is consistent with previous studies (Ye et al., 2013). Moreover, as shown in **Figure 3B**, Kaplan–Meier survival analysis showed that the overall survival time in NSCLC patients expressing high NOTCH3 were shorter. To verify these observations, we compared the protein levels of Notch3 in lung tissues of normal mice and lung cancer mice (*in vivo*), as well as in human bronchial epithelial cells (BEAS.2B) and NSCLC cells (A549 and H1299) (*in vitro*) using Western blot analysis, respectively. As expected, higher Notch3 levels were observed in tumor tissues and NSCLC cells compared to normal lung tissue and normal cells, respectively (**Figure 3C**).

### EVO Inhibited NOTCH3 Signaling in NSCLC Cells

**FIGURE 4:**
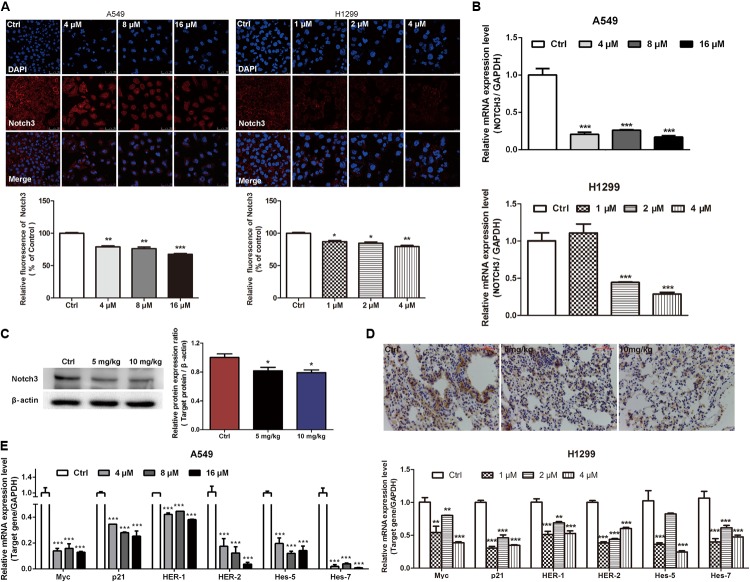
EVO inhibited NOTCH3 signaling *in vitro* and *in vivo*. **(A)** A549 and H1299 cells were treated with vehicle or EVO (4, 8, and 16 μM for A549 cells and 1, 2, and 4 μM for H1299 cells, respectively) for 48 h. NOTCH3 was detected by immunofluorescence assay. Cells were stained by an antibody recognizing Notch3 (red fluorescence), and the nuclei were visualized with the nuclear dye DAPI (blue fluorescence). Representative images and relative fluorescent levels were shown. **(B)** After 24 h treatment, total mRNA was extracted for NOTCH3 mRNA levels detection by using real-time PCR analysis. **(C)** Protein levels of Notch3 in vehicle- and EVO-treated groups were detected by Western blot analysis. Tissues in each group were mixed individually. **(D)** Representative photographs of immunohistochemical staining of NOTCH3 in the tissues of vehicle- and EVO-treated groups (5 mg/kg, 10 mg/kg). Tissues were immunohistochemically stained using an antibody against Notch3. **(E)** Total mRNA was extracted for NOTCH3 downstream gene (Myc, p21, HER-1, HER-2, hes-5, and hes-7) expression levels detection by using real-time PCR analysis. Data were presented as mean ± SD from three independent experiments, ^∗^*p* < 0.05, ^∗∗^*p* < 0.01, ^∗∗∗^*p* < 0.001, *vs.* vehicle.

It has been demonstrated that aberrant activation of NOTCH3 signaling is associated with the pathogenesis of NSCLC by favoring cancer cells survival, proliferation, invasion, migration, and stemness (Li et al., 2011; Hassan et al., 2016). Here, we investigated whether EVO modulates the constitutive NOTCH3 activation *in vitro* and *in vivo*. It was found that EVO dose-dependently reduced the NOTCH3 expression (**Figure 4A**) and the mRNA level of NOTCH3 (**Figure 4B**) in NSCLC cells. Moreover, we further determined the expressions of NOTCH3 in lung tumor tissues using Western blotting and immunohistochemistry. As shown in **Figures 4C,D**, levels of NOTCH3 were remarkably decreased in EVO-treated group compared to vehicle-treated group, which were consistent with our *in vitro* results (**Figures 4A,B**). Next, we determined the mRNA levels of NOTCH3 target genes. As expected, EVO significantly downregulated the mRNA levels of Myc, p21 (involved in cell cycle), HER-1, HER-2 (involved in cell growth), hes-5 and hes-7 (involved in cell differentiation) in both A549 and H1299 cells (**Figure 4E**).

### EVO Reversed NOTCH3 Hypomethylation in NSCLC Cells

**FIGURE 5:**
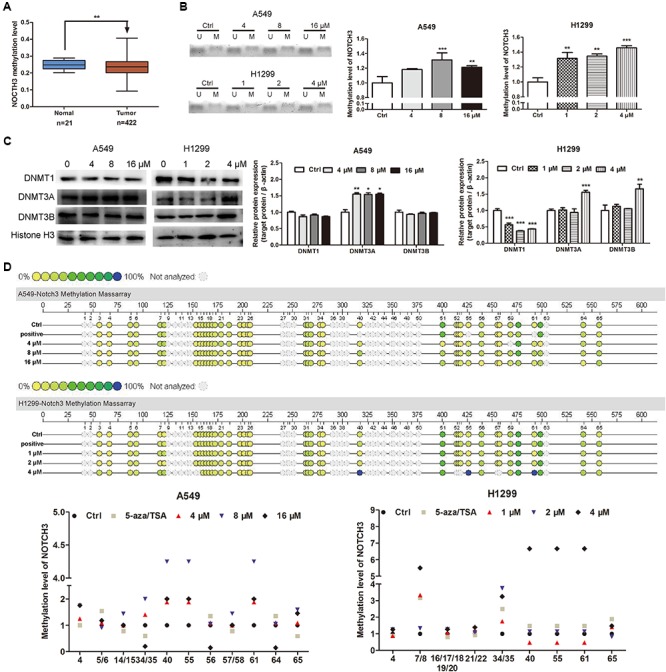
EVO induced NOTCH3 methylation in NSCLC cells. **(A)** NOTCH3 methylation level in the normal and lung tumor tissues were checked and analyzed in TCGA from MethHC database. The dataset includes 422 cases of lung cancer patients and 21 cases of normal persons. **(B)** The accurate content of NOTCH3 methylated DNA were detected by MeDIP-qPCR in A549 and H1299 cells. **(C)** Cells were treated with EVO (4, 8, and 16 μM for A549 cells and 1, 2, and 4 μM for H1299 cells, respectively) for 48 h. Protein levels of DNMTs in these two cell lines were detected by Western blot analysis. In **(B)** and **(C)**, Data were presented as mean ± SD from three independent experiments, ^∗^*p* < 0.05, ^∗∗^*p* < 0.01,^∗∗∗^*p* < 0.001, *vs.* vehicle. **(D)** Quantitative methylation profiling of NOTCH3 promoter in A549 and H1299 cells were analyzed by MassARRAY system. Each experiment is repeated and performed in triplicate, and mean value quantification is presented in lower panel.

Abnormal DNA methylation of oncogene is a major factor that leading to the tumor development and progression (Jones and Baylin, 2002; Yamamoto et al., 2011). Low DNA methylation levels (i.e., DNA hypomethylation) could enhance the formation of solid tumors, e.g., hepatocellular carcinoma, lung carcinoma and so on. As shown in **Figure 5A**, MethHC analyses demonstrated that the NOTCH3 methylation levels in human lung adenocarcinoma tissues were lower than that in human normal lung tissues. Since EVO downregulated the expressions of NOTCH3 in NSCLC cells (**Figures 4A,B**) and lung tumors (**Figures 4C,D**), here, we tried to explore whether EVO could affect NOTCH3 methylation. MSP assay indicated that compared with control, the methylation ratios of NOTCH3 (methylated/unmethylated) were markedly increased by 1.18-fold, 1.31-fold, and 1.21-fold in A549 cells after 4, 8, and 16 μM of EVO treatments, respectively. Similarly, EVO treatments also increased the methylation ratios of NOTCH3 by 1.32-fold, 1.35-fold, and 1.46-fold in H1299 cells compared with that in vehicle-treated group (**Figure 5B**). All these suggested that EVO could serve as an effective NOTCH3 epigenetic stimulator by increasing the DNA methylation level. To further explore the mechanisms of EVO-induced NOTCH3 methylation, the protein levels of classical epigenetic modifiers (DNMTs) were detected by using the Western blot analysis. DNMTs (DNMT1, DNMT3A and DNMT3B) are responsible for DNA methylation at the 5-position of cytosine (Rajendran et al., 2011). Compared with control, EVO increased the DNMT3A protein levels in A549 cells; 4 μM of EVO greatly increased the protein levels of DNMT3A and DNMT3B in H1299 cells (**Figure 5C**). To further determine the methylation site of NOTCH3, mass assay was carried out. As shown in **Figure 5D**, 15 CG methylation sites of NOTCH3 DNA promoter region was shown in A549 cells, among them, 5 CG sites of methylation level in EVO-treated group increased by 2-fold compared with control group; Similarly, among 16 CG methylation sites of NOTCH3 DNA promoter region in H1299 cells, 7 CG sites of methylation level in EVO-treated group was increased by 2-fold compared with control group. Taken together, EVO inhibited NOTCH3 signaling by activation of the DNMTs-induced NOTCH3 methylation.

### Inhibition of NOTCH3 Methylation in NSCLC Cells Diminished EVO’s Effects on Cell Viability, Cell Cycle Arrest and Stemness

**FIGURE 6:**
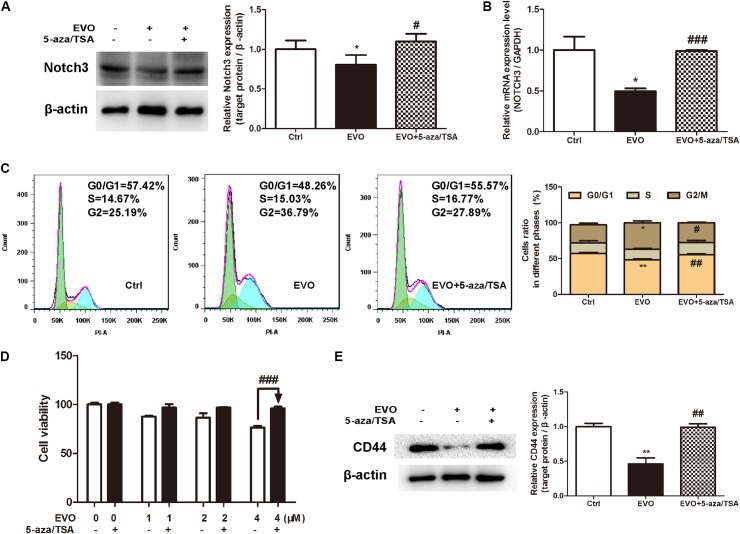
Inhibition of NOTCH3 methylation diminished EVO’s anti-lung cancer effects. **(A)** H1299 cells were treated with EVO (2 μM) in the presence or absence of 5-aza/TSA (2.5/0.5 μM) for 48 h. The protein levels of Notch3 in H1299 cells were detected by Western blot analysis. **(B)** Total mRNA was extracted by using real-time PCR analysis. **(C)** After fixation, cells were stained with PI and then analyzed using flow cytometry. Representative images (left) and the statistical analysis of cell cycle data in three independent experiments (right) were shown. **(D)** Cells were treated with different concentrations of EVO in the presence and absence of 5-aza/TSA (2.5/0.5 μM) for 48 h, cell viability was measured by the MTT assay. **(E)** Cells were treated as previously described in **(A)**, then cells were extracted for Western blot analysis by using antibody specific to CD44. Protein level of the vehicle-treated cells was regarded as 1. Data were presented as mean ± SD from three independent experiments, ^∗^*p* < 0.05, ^∗∗^*p* < 0.01 *vs.* vehicle; ^#^*p* < 0.05, ^##^*p* < 0.01, ^###^*p* < 0.001 *vs.* EVO.

To further determine whether EVO-inhibited NOTCH3 activation is dependent on DNMTs, a classic DNMTs inhibitor 5-aza combination with a histone deacetylase inhibitor TSA were used. Results showed that EVO decreased the protein and mRNA levels of NOTCH3 by 19.68% ± 2.48% and 49.75% ± 6.12%, respectively (**Figure 6A**). While, the protein and mRNA levels of NOTCH3 were increased by 29.87 ± 4.31% and 50.68% ± 1.18% after 5-aza/TSA (2.5/0.5 μM) intervention, respectively (**Figures 6A,B**). Furthermore, we found that combination with 5-aza/TSA (2.5/0.5 μM) diminished EVO’s effects on cell cycle arrest (**Figure 6C**), cell viability (**Figure 6D**) and stemness (**Figure 6E**).

## Discussion

Lung cancer, one of the major life-threatening cancer, is difficult to be detected and treated in early stage (Guo et al., 2015). Despite a wide variety of therapies are used for lung cancer treatment, the prognosis for lung cancer is still very poor. Molecular targeted therapy is an important therapeutic approach for NSCLC management. EGFR-TKIs and ALK/ROS1 inhibitors have been widely used in the clinical practice (Ge and Shi, 2015). EGFR can affect the tumor cell differentiation, proliferation, survival, adhesion, apoptosis, and migration (Cappuzzo et al., 2005). About 43–89% of NSCLC patients overexpressed EGFR in tissue samples. Patients who show high expression of EGFR are not sensitive to chemotherapy and radiation. Thus, EGFR was thought to be a prospective therapy target (Raymond et al., 2000). ALK is activated via three main mechanisms: overexpression, activating point mutations, and fusion protein expression. It is a key oncogenic driver in NSCLC (Capelletti et al., 2014). ROS1 rearrangement can be detected in 1.7–4.6% of NSCLC patients, and has been considered as a driver gene in NSCLC (Gainor and Shaw, 2013). Advanced NSCLC patients could benefit from EGFR-TKIs and ALK/ROS1 inhibitors, but the drug resistance will show up in the following year (Chong and Janne, 2013). Therefore, identification of the novel molecular targets for early detection and better prevention/treatment are urgently needed. The key to targeted therapy is to find effective targets. NOTCH3 is mainly expressed in NSCLC and has been shown to be a promising target of NSCLC.

Currently available chemotherapeutics against lung cancer have various disadvantages (Tong et al., 2016). Therefore, exploring effective and safe novel targeted therapeutic agents against NSCLC are needed. Utilizing food- or herb-derived natural products with tolerable adverse effects and broad effectiveness to prevent malignant tumor is a promising approach for improving the overall survival rate. EVO is one of the main bioactive constituents derived from *Euodiae Fructus*. Many biological and pharmacological activities including the anti-cancer activity that may provide health benefits to humans has been attributed to EVO (Lin et al., 2016). Many studies have reported that EVO exerted the inhibitory effects on lung tumor growth (Hong et al., 2014) and metastasis (Ogasawara and Suzuki, 2004). Nevertheless, the anti-NSCLC mechanisms of action of EVO are uncertain. In this study, we investigated the anti-NSCLC activity of EVO in human NSCLC cells and in urethane-induced lung cancer FVB mice. We found that EVO suppressed cell viability, induced G2/M cell cycle arrest, inhibited cell migration and reduced stemness in NSCLC cells (**Figure 2**), furthermore, EVO significantly inhibited the lung tumor size and tumor numbers (**Figure 1**). For the *in vivo* experiment, 5 and 10 mg/kg of EVO were used. Whether EVO treatment with higher dose exhibits more potent anti-lung cancer effects is a question to be addressed. It is noteworthy that i.g. 10 mg/kg (high dose) of EVO showed significant inhibitory effect on tumor size and tumor numbers in mice, and it did not cause observable reduction in the body weight of mice and any other abnormalities in clinical signs. Several other published studies also used the same drug dosage in mice for treating or preventing cancers including breast cancer (Liao et al., 2005; Du et al., 2013), colon cancer (Ogasawara et al., 2001), and so on. Hence, we chose a low but safe and effective dose of EVO for our *in vivo* study.

NOTCH3 signaling plays different roles in lung cancer cell lines. In the context of cell adhesion, motility and EMT, NOTCH3 shows opposite functions in NSCLC and SCLC. It acts as a tumor promoter in NSCLC but a tumor suppressor in SCLC. Regarding cell proliferation, inhibition of NOTCH3 suppresses cell proliferation and induces apoptosis in NSCLC, while, it shows no effect on SCLC cell proliferation (Hassan et al., 2016). These evidences support the fact that NOTCH3 signaling is highly dependent on cell type. In this study, we used two NOTCH3 activated NSCLC A549 and H1299 cell lines, and found that EVO exerts anti-NSCLC effects by inhibiting NOTCH3 signaling.

Studies suggest that alterations in DNA methylation is associated with several diseases including lung cancer (Li and Zhang, 2014). In mammals, global DNA methylation is mainly catalyzed by four DNMTs: DNMT1, DNMT2, DNMT3A, DNMT3B (Uysal et al., 2017). Abnormal expressions and activities of DNMTs are the cause of abnormal methylation of some oncogenes in tumor cells, which are the important factors for promoting the tumor progress and development. Even though all of them have highly conserved DNMT motifs, they show different functions. DNMT1, a maintenance DNMT, is critical for maintaining methylation patterns during DNA replication. DNMT2 methylates DNA at very low levels, its primary activity might be in RNA methylation (Hermann et al., 2004; Tovy et al., 2010). DNMT3A and DNMT3B are responsible for catalyzing *de novo* methylation of DNA during early embryogenesis (Okano et al., 1999). Both DNMT1 and DNMT3B genetic disruption were demonstrated to eliminate the activity of methyltransferase and thereby decrease DNA methylation by approximately 95% (Rhee et al., 2002). Based on the above reasons, we determined the protein levels of DNMTs (DNMT1, DNMT3A and DNMT3B) in NSCLC cells. Results showed that EVO up-regulated the DNMT3A protein level in NSCLC cells, and it also increased the protein level of DNMT3B in H1299 cells (**Figure 5C**), suggesting that activation of the DNMTs-induced NOTCH3 methylation may be one of the mechanisms for EVO’s anti-NSCLC effects. In addition, we found that EVO downregulated the protein level of DNMT1 in H1299 cells. How the DNMT1 protein level was inhibited by EVO treatment is a question to be elucidated in the future. Currently, the most widely used DNMT inhibitors are cytidine analogs like 5-azacytidine, 5-aza (decitabine) and pyrimidin-2-one ribonucleoside (zebularine). DNMT inhibitors have been employed to prevent and treat cancers through inhibiting aberrant DNA methylation and then activating silenced tumor suppressor genes. Studies have indicated that azacytidine and 5-aza have a strong demethylating effect, and they can re-express aberrantly silenced genes at a lower dose, which could reduce cell differentiation, proliferation and senescence. 5-aza has been approved by the United States Food and Drug Administration (FDA) for malignant tumor treatment. In this study, we used 5-aza as a positive control, and found that combination with 5-aza diminished EVO’s anti-lung cancer effects and the inhibition of NOTCH3 activation, suggesting that EVO may inhibit NOTCH3 signaling by activation of the DNMTs-induced NOTCH3 methylation.

Considering the multi-target nature of EVO, we believe that NOTCH3 is one of the targets of EVO. It is well known that the Notch signaling pathway has crosstalks with other pathways that are involved in oncogenesis, such as Wnt (Li et al., 2011), NF-kB (Clement et al., 2007), P53 (Giovannini et al., 2017) signaling pathways, and so on. In the future, other signaling pathways that are involved in the anti-NSCLC effects of EVO will be investigated.

## Conclusion

In summary, we demonstrate that a novel NOTCH3 methylation stimulator EVO exerts anti-lung cancer activities and inhibits NOTCH3 signaling. Our experiments show that EVO inhibits cell viability, induces G2/M cell cycle arrest, suppresses cell migration and reduces stemness in human NSCLC cells; further studies show that EVO potently inhibits NOTCH3 signaling by activation of DNMTs-induced NOTCH3 methylation. EVO’s effects on cell viability, cell cycle arrest and stemness can be diminished by DNMTs inhibitor. Moreover, EVO inhibits *in vivo* tumor growth in mice. These findings indicate that the anti-NSCLC activity of EVO is at least in part due to the inhibition of the NOTCH3 signaling pathway, suggesting that EVO is a promising candidate which can be developed as a preventive or therapeutic agent by targeting NOTCH3 signaling in NSCLC.

## Author Contributions

L-LL and Z-QL supervised the entire project and edited and finalized the manuscript. TS, XY, J-HD, and Q-JH performed the experiments, analyzed the data, and drafted the manuscript. S-CH, Y-MZ, H-MZ, and YW participated in performing the experiments. All authors read and approved the final manuscript.

## Conflict of Interest Statement

The authors declare that the research was conducted in the absence of any commercial or financial relationships that could be construed as a potential conflict of interest.

## Footnotes

1 http://maplab.imppc.org/wanderer/

2 www.kmplot.com

## Supplementary Material

FIGURE S1 Organ indexes of heart, liver, lung, kidney, spleen, and thymus. The index of each organ in vehicle- and EVO-treated groups were represented as: organ weight/body weight.

FIGURE S2 EVO reduced the proteins levels of cell cycle- and EMT-related molecules. H1299 cells were treated with EVO (1, 2, and 4 μM) for 48 h. The protein levels of cell cycle-related molecules (cdc2 and cyclin B1) **(A)**, and EMT-related molecules (E-cadherin, N-cadherin, and vimentin) **(B)** were determined by Western blotting. Data were presented as mean ± SD from three independent experiments, ^∗∗∗^*p* < 0.001, *vs.* vehicle.

TABLE S1 Sequence of PCR primers.
