# Supplementary figures and images for: Evodiamine, a Novel NOTCH3 Methylation Stimulator, Significantly Suppresses Lung Carcinogenesis in Vitro and in Vivo
Source: Front Pharmacol. 2018 May 1;9:434. doi: 10.3389/fphar.2018.00434 (PMC5938359; doi:10.3389/fphar.2018.00434)

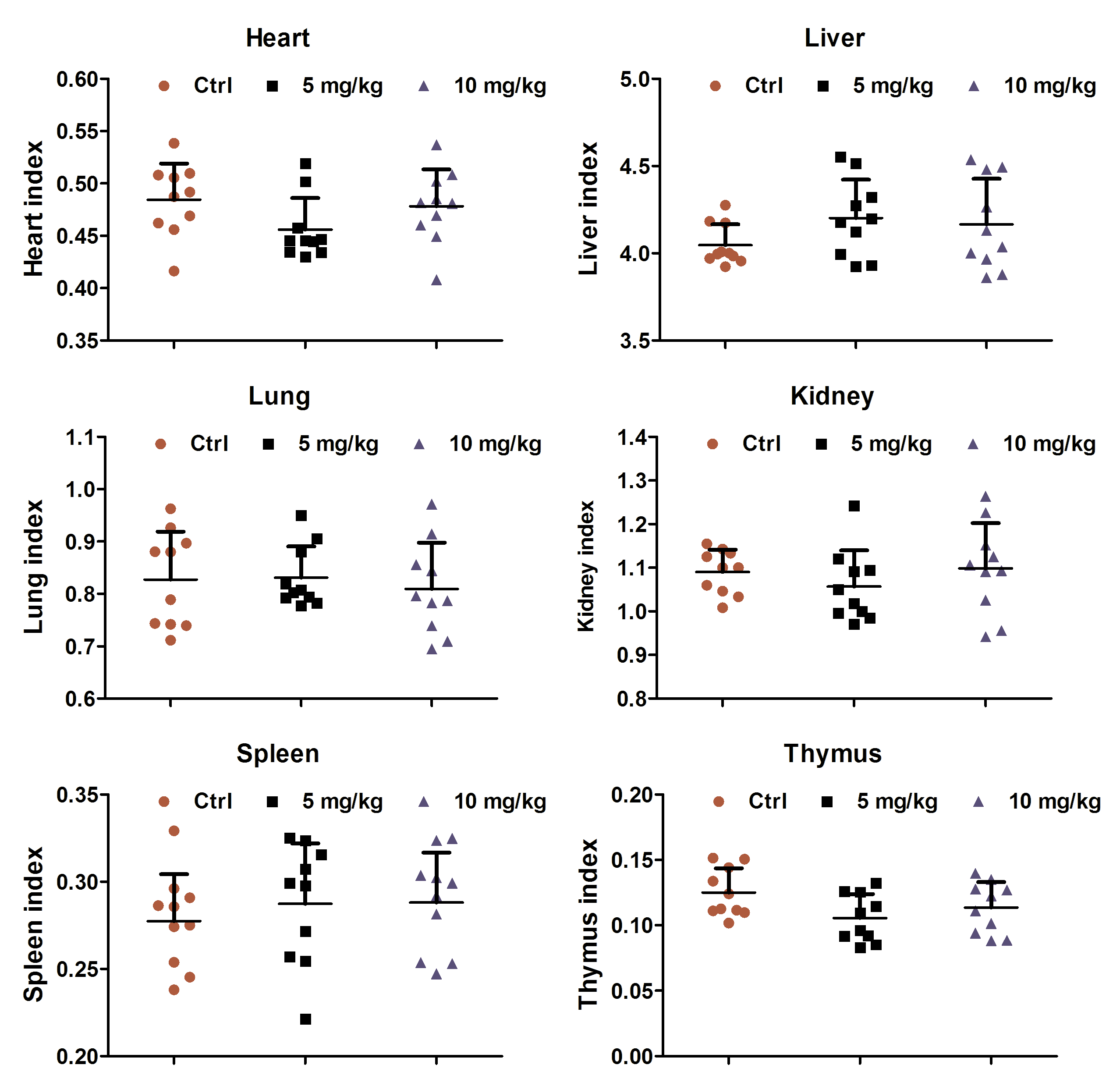

Supplement: FIGURE S1 — Organ indexes of heart, liver, lung, kidney, spleen, and thymus. The index of each organ in vehicle- and EVO-treated groups were represented as: organ weight/body weight. [file Image_1.TIF]

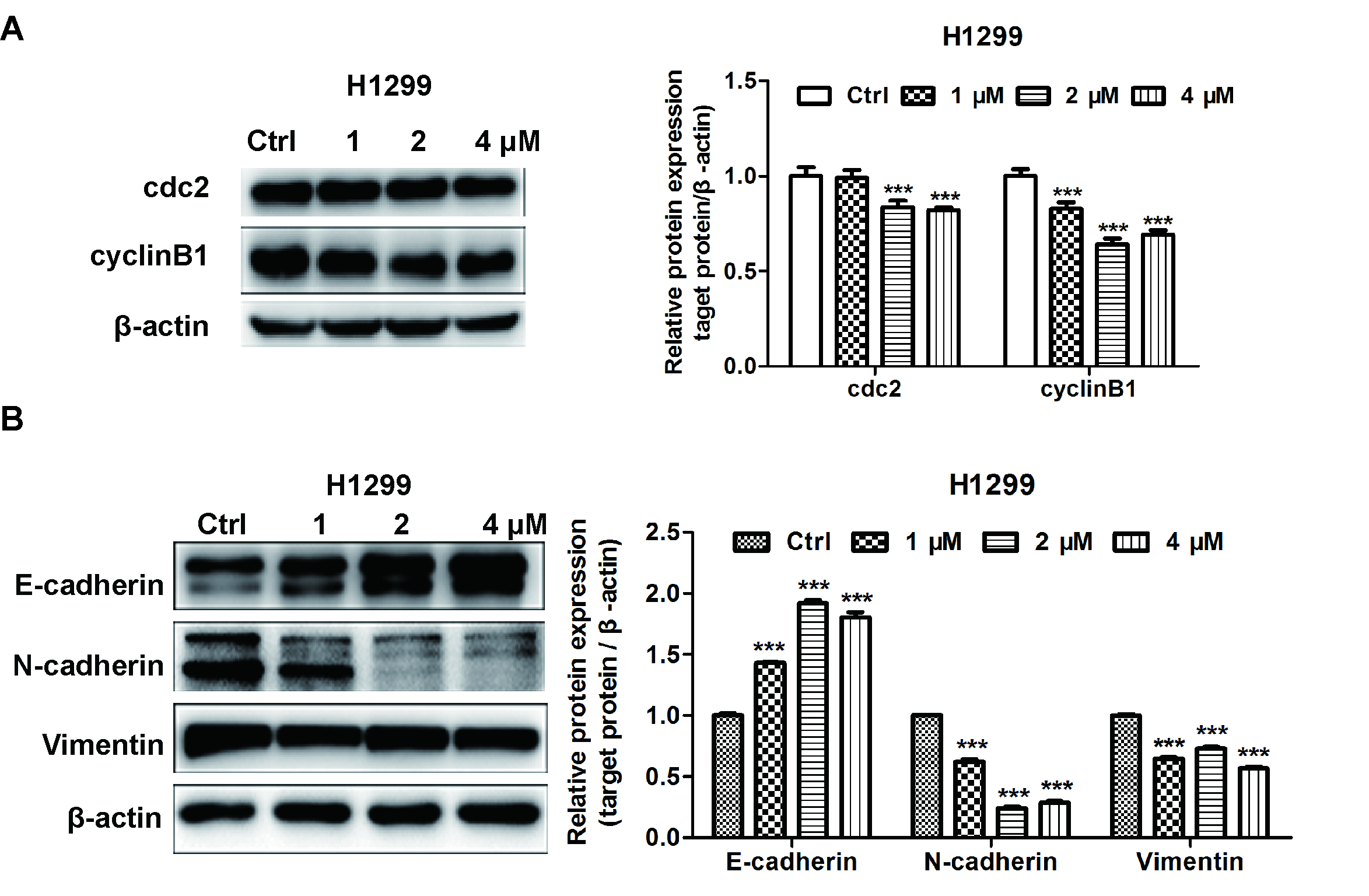

Supplement: FIGURE S2 — EVO reduced the proteins levels of cell cycle- and EMT-related molecules. H1299 cells were treated with EVO (1, 2, and 4 μM) for 48 h. The protein levels of cell cycle-related molecules (cdc2 and cyclin B1) (A), and EMT-related molecules (E-cadherin, N-cadherin, and vimentin) (B) were determined by Western blotting. Data were presented as mean ± SD from three independent experiments, ∗∗∗p < 0.001, vs. vehicle. [file Image_2.TIF]
