# Supplementary material for: Evodiamine, a Novel NOTCH3 Methylation Stimulator, Significantly Suppresses Lung Carcinogenesis in Vitro and in Vivo
Source: Front Pharmacol. 2018 May 1;9:434. doi: 10.3389/fphar.2018.00434 (PMC5938359; doi:10.3389/fphar.2018.00434)
Supplement: TABLE S1 — Sequence of PCR primers. [file Table_1.DOC]

**SUPPLEMENT TABLE S1. Sequences of PCR primers**

| Gene name | forward primers | reverse primers |
| --- | --- | --- |
| GAPDH | GGCCTCCAAGGAGTAAGACC | AGGGGAGATTCAGTGTGGTG |
| HER-1 | GCCTCCAGAGGATGTTCAATAA | TGAGGGCAATGAGGACATAAC |
| HER-2 | ACCTGCTGAACTGGTGTATG | TGACATGGTTGGGACTCTTG |
| Hes-5 | GTGCCTCCACTATGATCCTTAAA | CTTCCACGTGACTGAGAGTTC |
| Hes-7 | TACGGGTACTAGAGTGGGAATG | TCTCTCTCACCTGGCTAACA |
| P21 | ATCTTTCTAGGAGGGAGACACT | TCCTTGTTCCGCTGCTAATC |
| Myc | CATACATCCTGTCCGTCCAAG | GAGTTCCGTAGCTGTTCAAGT |
| Methylation NOTCH3 | TTTAGTTTTCGAAGGTTCGTTTC | CTACATACTCGACCTAAATTTCCGA |
| Unmethylation NOTCH3 | TTTTTAGTTTTTGAAGGTTTGTTTT | TCACTACATACTCAACCTAAATTTCCA |
